# Effects of *Bacillus cereus* and coumarin on growth performance, blood biochemical parameters, and meat quality in broilers

**DOI:** 10.14202/vetworld.2020.2484-2492

**Published:** 2020-11-23

**Authors:** Galimzhan Duskaev, Shamil Rakhmatullin, Olga Kvan

**Affiliations:** Department for Feeding Agricultural Animals and Fodder Technology, Federal Research Centre of Biological Systems and Agrotechnologies of the RAS, Orenburg, Russia

**Keywords:** *Bacillus cereus*, blood, broilers, coumarin, muscles, productivity

## Abstract

**Background and Aim:**

Progressive antibiotic resistance has become the primary threat to public health. The search for alternative substances with similar effects is now a global challenge for poultry farming. The aim of this study was to investigate the action of the probiotic *Bacillus cereus* (BC) and coumarin (CO) on broiler productivity, biochemical indicators of blood, and muscular and liver tissues.

**Materials and Methods:**

The trial of this study included Arbor Acres cross broiler chickens that were grown up to the age of 42 days. The experiment was conducted on 200 broiler chickens divided into four experimental groups of 50 individuals each: The control group received ration without additives (main ration [MR]), the first experimental group received MR+BC, the second received MR+CO, and the third received −MR+BC+CO. A biochemical and hematological analyzer was used to estimate elemental concentrations using inductively coupled plasma mass spectrometry and inductively coupled plasma atomic emission spectrometry.

**Results:**

Inclusion of CO and CO+BC in the diet improved growth rates and reduced feed consumption (FC) per kg of live weight gain. Decreased white blood cell count, increased creatinine and triglycerides (CO), changes in aminotransferase and transpeptidase activity, and increases in chemical elements in the liver and pectoral muscles (BC+CO) were observed. The inclusion of BC+CO in the diet contributed to increases in a greater number of chemical elements in the liver (calcium [Ca], K, magnesium, Mn, Si, and Zn) and the pectoral muscles (Ca, Na, Co, Cu, Fe, Mn, Ni, and Zn).

**Conclusion:**

The inclusion of CO and CO+BC in the diet improves growth rates and reduces FC in broilers against a background of the absence of mortality during the experiment.

## Introduction

Poultry production contributes significantly to food security in many countries worldwide. Previously, subtherapeutic dosages of feed antibiotics were widely used as growth stimulants to maintain livestock health and increase productivity. However, progressive antibiotic resistance has become a substantial threat to public health [1,2]. The search for alternative substances with similar effects is a global challenge to poultry farming [3,4]. In particular, plant substances, including medicinal plants, are of substantial interest. For example, the addition of *Phoenix dactylifera* seeds to the diet causes significant differences (p<0.05) in the relative growth rate, feed conversion rate, energy efficiency, antioxidant status, and immunity of broiler chickens [5]. Phenolic extracts from blueberries (*Vaccinium corymbosum*) and blackberries (*Rubus fruticosus*) have been evaluated as alternatives for broilers, and they modulate the intestinal microbiome of broilers and increase body weight (BW) gain [6].

Notably, extracts contain a significant number of various biologically active substances: *V. corymbosum* and *R. fruticosus* – cinnamic acid, coumarin (CO), ellagic acid, and gallic acid; *P. dactylifera* – 4,6-dimethyl-3-(4-methoxyphenyl) CO (14.73%), 4-methylcinnamic acid (11.44%), and 6-hydroxy-7-methoxycoumarin (8.71%) [7]. Therefore, it is essential to study these substances separately to find an alternative to antibiotics. CO is considered especially interesting: More than 1000 COs have been recorded from the seeds, roots, and leaves of plants with antioxidant, anticancer, anti-inflammatory, and antimicrobial properties [8,9].

The potential role of COs as alternative therapeutic strategies based on their ability to block quorum sensing signaling systems and inhibit the biofilm formation of clinically significant pathogens has been emphasized [10,11]. The presence of CO in food is not considered to pose a risk to humans [12,13]. Notably, CO is a new agricultural antibacterial substance against *Ralstonia solanacearum* [14]. In addition, new derivatives of CO, such as from the bark of *Zanthoxylum avicennae*, may have different effects [15,16]. Studying the additional effects of CO in interaction with other biologically active substances is no less interesting. The presence of synergistic mechanisms of Radix Angelicae containing COs and ligustrazin has been discovered in the treatment of migraines [17].

In our opinion, it is equally important to study possible synergistic effects with probiotic substances. It is known that *Bacillus* spp. are active against *Clostridium perfringens*, *Staphylococcus aureus*, and *Listeria monocytogenes* in poultry [18,19] and humans [20], and they positively affect the birds’ growth gain [21-24].

The aim of this study was to investigate the separate and joint action of the probiotic *Bacillus cereus* (BC), with anti-quorum activity [25], and CO derived earlier from an oak bark extract [26] on broiler productivity, biochemical indicators of blood, and muscular and liver tissues.

## Materials and Methods

### Ethical approval

The study included 1-day-old Arbor Acres cross broiler chickens grown up to the age of 42 days. Poultry maintenance and procedures during the experiment met the instructions and recommendations of Russian regulations (Order of the Ministry of Health of the USSR No. 755 of 12.08.1977) and the Guide for Care and Use of Laboratory Animals (National Academy Press, Washington, D.C., 1996).

### Study period and location

The study was performed in December-2019 at the Experimental Biological Clinic (vivarium) (Federal Research Centre of Biological Systems and Agrotechnologies of the RAS, 2019).

### Animals

The broilers were grown in group cages in a room with controlled temperature and humidity, and they had free access to food and water [27]. The experiment was conducted on 200 broiler chickens divided into four experimental groups with five replications of 10 individuals in each. During each of the three feeding phases, the birds were given the rations as presented in Table-1.

**Table-1:** Dietary ingredients (natural substance) and chemical composition (dry matter) of basal diets.

Ingredient composition (%)	Starter (1-21 days)	Grower (21-35 days)	Finisher (35-42 days)
		
	Control, I, II, III	Control, I, II, III	Control, I, II, III
Wheat	47.0	44.5	42.0
Barley	2.6	1.45	0.3
Corn	7.5	14.75	22.0
Soybean meal (46% protein)	25.0	20.0	15.0
Sunflower meal (38% protein)	7.0	8.5	10.0
Sunflower oil	5.0	5.0	5.0
Dicalcium phosphate	1.6	1.5	1.4
Mel stern	0.9	1.2	1.5
Limestone	0.5	0.4	0.3
Salt	0.36	0.28	0.2
DL-methionine	0.18	0.17	0.16
L-lysine	0.35	0.26	0.17
Vitamin-mineral premixa^a^	2.0	2.0	2.0
Calculated nutrients metabolizable energy (kcal g-1)	296.0	299.0	302.0
Crude protein	22.0	20.3	18.7
Methionine+cysteine	0.87	0.83	0.79
Lysine	1.35	1.15	0.96
Calcium	0.95	0.98	1.01
Available phosphorus	0.54	0.51	0.48

a=Supplied following per kilogram of diet (natural substance): Vitamin A=7000 IU, Vitamin D3=800.0 IU, Vitamin E=9 IU, Vitamin K3=1.1 mg, Thiamine=0.7 mg, Riboflavin=3.0 mg, Vitamin B6=1 mg, Vitamin B12=0.01 mg, Vitamin C=50 mg, Mn=23 mg, Fe=17 mg, Zn=11 mg, Cu=2.5 mg, I=0.4 mg, Se=0.2 mg

The control group received the main ration (MR), the first experimental group received MR+BC at a dose of 12.6×10^3^ microbial bodies/kg of feed/day, the second group received MR+CO at a dose of 2 mg/kg of feed/day, and the third received MR+BC+CO.

### Substances

1. BC IP 5832 (ATCC 14893).
2. CO – IUPAC: 7-hydroxycoumarin (Aldrich).

### Bird productivity and sample collection

At the end of each period, the BW of the broilers was assessed, and at the end of the experiment, feed consumption (FC) and mortality were determined.

BW gain (BWG) and FC per kg of live weight (FC) were calculated for each group. At the end of the experiment, 10 birds aged 42 days with average BW were selected, blood samples were intravitally taken from their wing veins, and then, the broilers were slaughtered. Samples of 50 g of the liver and muscular tissues were taken from the thoracic and thigh muscles from each bird, placed in a sterile container, and kept at −20°C.

### Laboratory analysis

Hemoglobin (Hb), hematocrit (Ht), red blood cell count (RBC), and white blood cell count (WBC) were determined using a URIT-2900 Vet Plus hematological analyzer (URIT Medical Electronic Group Co., Ltd., China, 101040000000086).

Diagnostic kits developed by Diakon-DS (Russia) and Ransod by Randox were used to determine the content of biochemical indicators in the blood plasma samples: Glucose (GLU), total protein, albumin (ALB), uric acid (UA), urea (UREA), bilirubin (BIL), creatinine (CREAT), total cholesterol (TC) and its fractions, that is, high-density cholesterol (HDL), and triacylglycerols (TGs). Enzyme activity was determined in a CS-T240 automatic biochemical analyzer (DIRUI Industrial Co., Ltd., China, 0101320001): Alkaline phosphatase (ALP; EC 3.1.3.1), alanine aminotransferase (ALT; EC 2.6.1.2), aspartate aminotransferase (AST; EC 2.6.1.1), and lactate dehydrogenase (LDH; EC 1.1.1.27); phosphorus (P), calcium (Ca), and magnesium (Mg).

Analysis of the elemental composition of substrates was conducted at the Center for Biotic Medicine laboratory (Moscow) and included an assessment of concentrations of elements: Ca, K, Mg, Na, P, As, B, Co, Cr, Cu, Fe, I, Li, Mn, Ni, Se, Si, V, and Zn using mass spectrometry with inductively coupled plasma (MS-ICP) and atomic emission spectrometry with inductively coupled plasma (AES-ICP) using a Nexion 300D quadrupole mass spectrometer (PerkinElmer, USA) and an Optima 2000 DV atomic emission spectrometer (PerkinElmer, USA). A Multiwave 3000 microwave decomposition system (Anton Paar, Austria) was used for ashing.

### Statistical analysis

Statistical analysis was conducted using SPSS Statistics Version 20 (IBM, USA) program, which was used to calculate the average value (M) and standard deviation error (m), and results were considered significant at p<0.05.

## Results

### The effect of BC and CO on broiler chicken productivity

The inclusion of CO in the diet, as well as CO+BC, contributed to an increase in the live weight of broilers, especially from the 28^th^ to the 42^nd^ days of the experiment (CO by 15.2-20.8%, p<0.05; CO+BC by 15.6-12.8%, p<0.05). There was a lower FC in animals fed with CO (p<0.05) against a background of the absence of deaths during the entire period of the experiment (Table-2).

**Table-2:** The effect of *Bacillus cereus* and coumarin on broiler chicken productivity.

Groups	BW (kg)					FC 1-42 days	Mortality rate (%)

	7 days	21 days	28 days	35 days	42 days		
Control	0.214±6.2	0.707±57.8	1.215±91.1	1.768±45.7	2.388±70.5	1.65	2
BC	0.217±6.0	0.741±27.2	1.297±47.0	1.896±37.7	2.605±73.8	1.58	1
CO	0.219±5.7	0.783±19.9	1.400±57.2	2.139±40.9^a^	2.886±42.6^a^	1.49^a^	0
BC+CO	0.219±5.5	0.786±17.8	1.371±31.0	2.044±44.5^a^	2.694±50.0^a^	1.59	0

BW=Body weight, FC=Feed consumption. a=Means with letters within a row are significantly different at p≤0.05

### Blood parameters with BC and CO in diets

The separate use of BC in the broilers’ diet led to a decreased WBC (p<0.05) compared to other groups against a background of higher values for granulocytes and Ht. In addition, joint feeding (CO+BC) increased the values of granulocytes (p<0.05), Hb, and Ht (Table-3).

**Table-3:** Morphological blood parameters with *Bacillus cereus* and coumarin in diets (day 42).

Indicator	Control	BC	CO	BC+CO
WBC, 109 cell/l	59.17±2.07	47.90±0.67^a^	59.67±5.87	55.27±2.38
LYM, 109 cell/l	56.63±4.43	51.73±1.33	54.40±5.72	52.20±1.27
MON, 109 cell/l	8.30±0.10	8.50±0.45	7.30±1.05	7.80±0.40
GR, 109 cell/l	35.07±4.33	45.43±6.54^a^	38.57±4.41	45.87±6.50^a^
RBC, 10^12^ cell/l	3.97±0.01	3.89±0.02	3.51±0.13	3.59±0.29
Hb, g/l	99.67±2.84	113.67±1.20	113.0±13.11	116.0±3.51
Ht, %	18.23±3.77	21.03±0.41	20.07±2.69	21.10±0.86
PLT, 10^9^ cell/l	101.67±11.86	102.33±6.17	109.3±6.96	116.0±4.62

WBC=Leukocytes, LYM=Lymphocytes, MON=Monocytes, GR=Granulocytes, RBC=Erythrocytes, Hb=Hemoglobin, Ht=Hematocrit, PLT=Platelets, a=Means with letters within a row are significantly different at p≤0.05

The inclusion of CO in the diet reduced the content of GLU in the serum of broilers (p<0.05) as well as that of cholesterol (p<0.05), and it increased TG (p<0.05) similarly to BC, CREAT (p<0.05), UA (p<0.05), and Fe (p<0.05) (Table-4).

**Table-4:** Biochemical blood parameters with *Bacillus cereus* and coumarin in diets (day 42).

Indicator	Control	BC	CO	BC+CO
GLU, mmol/l	13.94±0.49	13.19±0.32	9.19±3.53a	13.97±0.65
TP, g/l	38.00±1.40	37.05±3.01	36.34±6.11	37.69±0.86
ALB, g/l	12.67±0.33	11.67±0.67	11.33±2.03	12.0±0.58
BIL, mcmol/l	0.36±0.02	0.34±0.03	0.30±0.05	0.29±0.03^a^
TC, mmol/l	3.22±0.37	2.96±0.16	2.88±0.26^a^	3.75±0.29
TG, mmol/l	0.21±0.08	0.67±0.49^a^	0.66±0.12^a^	0.34±0.17
CREAT, mcmol/l	111.43±6.03	121.90±10.64	124.23±9.05^a^	119.57±9.05
UREA, mmol/l	3.27±0.09	3.20±0.06	3.13±0.09	3.23±0.13
UA, mcmol/l	188.7±27.83	214.0±12.28	387.6±198.73^a^	249.7±78.01^a^
Fe, mcmol/l	16.8±1.29	15.53±1.11	19.53±3.64^a^	19.07±1.13^a^
P, mmol/l	2.05±0.11	2.92±0.43	2.98±0.44	3.75±1.11a

GLU=Glucose, TP=Total protein, ALB=Albumin, BIL=Bilirubin, TC=Cholesterol, TG=Triacylglycerol, CREAT=Creatinine, UREA=Urea, Fe=Iron, P=Phosphorus, UA=Uric acid. a=Means with letters within a row are significantly different at p≤0.05

The joint use of BC+CO reduced only BIL (p<0.05) against a background of increasing UA and minerals (Fe, P).

The activity of the endogenous enzyme ALT in the CO group was higher (p<0.05) in comparison with other groups (Table-5).

**Table-5:** The activity of serum enzymes with *Bacillus cereus* and coumarin in diets (day 42).

Indicator	Control	BC	CO	BC+CO
ALT, unit/l	12.23±0.95	18.0±1.67	25.1±6.14^a^	18.13±2.51
AST, unit/l	88.80±60.93	27.70±12.73^a^	42.60±24.68^a^	28.17±18.53^a^
GGT, unit/l	83.30±8.34	90.37±12.34	66.60±7.16^a^	69.87±5.95^a^
ALP, unit/l	243.67±10.75	194.93±35.9	196.87±9.99	241.20±20.99

ALT=Alanine aminotransferase, AST=Aspartate aminotransferase, GGT=Gamma-glutamyl transpeptidase, ALP=Alkaline phosphatase, a=Means with letters within a row are significantly different at p≤0.05

The activity of AST in all experimental groups decreased significantly (p<0.05) as well as γ-glutamyltransferase in groups with CO (p<0.05).

### The concentration of chemical elements in broiler

The inclusion of BC and CO in the diet contributed to increases in chemical elements in the liver tissue: Ca (p<0.05) except CO; K (p<0.05) and Mg (p<0.05) except BC and CO; As (p<0.05), B (p<0.05), Co (p<0.05), Cr, and I (p<0.05) except BC+CO; Fe (p<0.05) in the BC group; Mn (p<0.05) in the BC+CO group; Ni (p<0.05) except CO and BC+CO; Si (p<0.05) and Zn (p<0.05) in BC+CO; and a decrease of Li and V (Table-6).

**Table-6:** The concentration of chemical elements in broiler liver with *Bacillus cereus* and coumarin in diets (day 42).

Element*	Control	BC	CO	BC+CO
Ca	0.22±0.023	0.27±0.028^a^	0.25±0.025	0.28±0.028^a^
K	13.56±1.356	15.56 ±1.556	15.7±1.578	16.03±1.604^a^
Mg	0.76±0.076	0.86±0.086	0.88±0.088	0.95±0.096^a^
Na	2,47±0.247	3.10±0.311	3.88±0.389	3.36±0.336
P	12.74±1.274	14.45±1.446	14.22±1.422	14.81±1.481
As	0.023±0.003	0.035±0.005^a^	0.038±0.006^a^	0.027±0.004^a^
B	1.52±0.150	1.80±0.180^a^	1.74±0.170^a^	1.69±0.170^a^
Co	0.047±0.007	0.053±0.008^a^	0.050±0.007^a^	0.053±0.008^a^
Cr	0.365±0.044	0.489±0.059^a^	0.268±0.032^a^	0.370±0.044
Cu	20.65±2.070	23.11±2.310	23.30±2.330	21.62±2.160
Fe	702.0±70.0	1 461.0±146.0	1 127.0±113.0	1 342.0±134.0
I	0.86±0.104	1.08±0.110^a^	0.942±0.113	0.862±0.103^a^
Li	0.13±0.016	0.108±0.013	0.049±0.007^a^	0.059±0.009a
Mn	15.3±1.530	15.04±1.500	16.44±1.640	18.8±1.880
Ni	0.036±0.005	0.202±0.024^a^	0.01±0.002^a^	0.05±0.008^a^
Se	3.41±0.340	3.49±0.350	3.85±0.390	4.05±0.400
Si	3.87±0.390	6.15±0.620^a^	7.03±0.700^a^	5.96±0.600^a^
V	0.13±0.016	0.063±0.009^a^	0.06±0.009^a^	0.088±0.013^a^
Zn	88.76±8.88	102.0±10.0	103.0±10.0	117.0±12.0^a^

* Ca, K, Mg, Na, P-g/kg, As, B, Co, Cr, Cu, Fe, I, Li, Mn, Ni, Se, Si, V, Zn-mg/kg. a=Means with letters within a row are significantly different at p≤0.05

The inclusion of BC in the diet contributed to increases in chemical elements in the thoracic muscles: Co (p<0.05), Ni (p<0.05), and Zn (p<0.05); CO to increases in Ca (p<0.05), Na (p<0.05), Co (p<0.05), and Ni (p<0.05); and BC+CO to increases in Ca, Na, Co, Cu, Fe, Mn, Ni, and Zn (Table-7).

**Table-7:** Concentrations of chemical elements in the thoracic muscles of broilers with *Bacillus cereus* and coumarin in diets (day 42).

Element*	Control	BC	CO	BC+CO
Ca	0.16±0.017	0.16±0.017	0.19±0.02^a^	0.19±0.02^a^
K	20.98±2.099	19.13±1.913	20.84±2.085	19.51±1.951
Mg	1.32±0.132	1.35±0.136	1.35±0.136	1.33±0.133
Na	1.70±0.17	1.58±0.158	2.41±0.241a	2.18±0.218^a^
P	11.19±11.20	10.97±1.097	10.16±1.016	11.07±1.108
As	0.033±0.005	0.021±0.003^a^	0.016±0.002^a^	0.024±0.004^a^
B	1.89±0.19	1.66±0.17^a^	0.83±0.1^a^	1.86±0.19
Co	0.003±0.001	0.004±0.001^a^	0.018±0.003^a^	0.011±0.002^a^
Cr	0.203±0.024	0.15±0.019^a^	0.108±0.013^a^	0.21±0.025
Cu	2.08±0.21	1.73±0.17^a^	1.53±0.15^a^	3.32±0.33^a^
Fe	30.55±3.060	27.98±2.800	27.19±2.72^a^	41.27±4.130
I	1.6±0.160	0.79±0.096^a^	0.30±0.036^a^	0.79±0.095^a^
Li	0.022±0.003	0.009±0.002^a^	0.30±0.037^a^	0.008±0.002^a^
Mn	0.69±0.083	0.57±0.069	0.66±0.08	0.74±0.089
Ni	0.022±0.003	0.034±0.005^a^	0.033±0.005^a^	0.04±0.006^a^
Se	0.77±0.093	0.66±0.08^a^	0.48±0.059^a^	0.83±0.101^a^
Si	10.47±1.05	7.28±0.73^a^	6.02±0.6^a^	9.67±0.97
V	0.02±0.003	0.014±0.002^a^	0.008±0.002^a^	0.019±0.003^a^
Zn	16.06±0.61	24.77±0.48^a^	21.73±2.17^a^	30.95±3.09^a^

* Ca, K, Mg, Na, P-g/kg, As, B, Co, Cr, Cu, Fe, I, Li, Mn, Ni, Se, Si, V, Zn-mg/kg. a=Means with letters within a row are significantly different at p≤0.05

Notably, this increase occurred against a background of a decline in some substances: As (p<0.05), I (p<0.05), Li (p<0.05), Si (p<0.05), and V (p<0.05); in BC and CO groups – B (p<0.05), Cr (p<0.05), Cu (p<0.05), and Se (p<0.05). The inclusion of BC, CO, and BC+CO in the diet contributed to a decline in most chemical elements in the thigh muscles except for K, Mg, P, and B, the level of which hardly changed (Table-8).

**Table-8:** Concentrations of chemical elements in the thigh muscles of broilers with *Bacillus cereus* and coumarin in diets (day 42).

Element*	Control	BC	CO	BC+CO
Ca	0.546±0.055	0.181±0.018^a^	0.278±0.028^a^	0.181±0.018^a^
K	18.953±1.895	18.882±1.888	18.445±1.845	18.234±1.823
Mg	1.170±0.117	1.303±0.130	1.207±0.121	1.169±0.117
Na	3.000±0.300	2.141±0.214^a^	2.813±0.281	2.846±0.285
P	9.479±0.948	10.167±1.017	10.366±1.037	9.370±0.937
As	0.022±0.003	0.018±0.003^a^	0.021±0.003	0.020±0.003
B	0.997±0.120	0.918±0.110	0.870±0.104^a^	0.943±0.113
Co	0.010±0.002	0.006±0.001^a^	0.007±0.001^a^	0.007±0.001^a^
Cr	0.147±0.018	0.064±0.010^a^	0.074±0.011^a^	0.115±0.014^a^
Cu	2.840±0.280	1.490±0.150^a^	2.160±0.220^a^	2.380±0.240
Fe	61.300±6.130	26.400±2.640^a^	33.240±3.320^a^	36.630±3.660^a^
I	0.381±0.046	0.240±0.029^a^	0.128±0.015^a^	0.313±0.038^a^
Li	0.151±0.018	0.071±0.011^a^	0.020±0.003^a^	0.046±0.007^a^
Mn	0.873±0.105	0.589±0.071^a^	0.631±0.076^a^	0.728±0.087^a^
Ni	0.392±0.047	0.047±0.007^a^	0.111±0.013^a^	0.077±0.012^a^
Se	0.732±0.088	0.553±0.066^a^	0.526±0.063^a^	0.567±0.068^a^
Si	7.290±0.730	4.440±0.440^a^	2.540±0.250^a^	5.700±0.570^a^
V	0.015±0.002	0.010±0.002	0.010±0.002	0.011±0.002
Zn	82.620±8.260	27.420±2.740^a^	56.870±5.690^a^	53.190±5.320^a^

* Ca, K, Mg, Na, P-g/kg, As, B, Co, Cr, Cu, Fe, I, Li, Mn, Ni, Se, Si, V, Zn-mg/kg. a=Means with letters within a row are significantly different at p≤0.05

## Discussion

The inclusion of BC, CO, and CO+BC in the diet contributed to an increase in the live mass of broiler chickens: lower FC in animals was noted against a background of the absence of bird deaths during the entire period of the experiment. There are no records of the use of CO for poultry, but our findings indirectly confirm the conclusions of El-Far *et al*. [5] who added *P. dactylifera* seeds containing more than 25% of CO derivatives as well as Gong *et al*. [28] who used the probiotic BC in rations for broilers.

It is likely that the positive effect on the growth of broilers fed with CO and BC+CO could be related to previously discovered antimicrobial properties of BC [18], and similar properties of CO [10], which are based on their ability to block the QS signaling systems in BC and to inhibit the formation of biofilms of clinically significant pathogens. At a concentration of 50 μg/mL, CO inhibits the formation of *E. coli* biofilm by more than 80% without influencing bacterial growth [12]. This activates beneficial microflora, more complete fission, and absorption of substances and increases the productivity of poultry [29], which was confirmed in this experiment.

The previous studies have shown that the use of biologically active substances extracted from plant products (analog of gallic acid, phenolic compounds including CO) within 6 weeks allows 9.5% more BWG, and the microbiome of broiler chickens has a higher ratio of Firmicutes to Bacteroidetes [6,30]. Moreover, the previous studies reported that the growth-stimulating effects of probiotics in poultry are associated with a decrease in the amount and diversity of the natural microbiota, which increases the use of nutrients by the host’s intestinal epithelial cells and reduces exposure to harmful microbial metabolites [21].

In particular, depending on the strain, BC can grow in the form of submerged or floating biofilms and secrete a wide range of metabolites and enzymes in biofilm [31]. Moreover, the screening of differentially expressed immune-related genes from the spleen of broilers receiving the probiotic BC shows that they could play an important role in improving the immunity of broilers [32].

In this experiment, the synergistic effect of CO+BC on growth was less pronounced, as opposed to the individual addition of CO to the broiler diet.

The separate use of BC in the diet of broilers led to a decrease in WBC compared with other groups against a background of higher values for granulocytes and Ht. In our opinion, the change in blood indicators depends on the dosage of the probiotic substance. It is well known that a 1×10^6^ CFU/g dosage of *Bacillus licheniformis* and *Bacillus subtilis* contributes to an increase in RBC, WBC, and Hb along with a decline of AST, ALT, and ALP in experimental fish [33]. In addition, another study using *Bacillus clausii* showed that WBC values vary with increasing Ht [34].

Notably, the data are consistent with previous experiments on broiler chickens given feed with the addition of a probiotic preparation in combination with vitamins and minerals (BC, *B. licheniformis*, and *Bacillus megaterium*), where a lower WBC and number of eosinophils (p≤0.05) were found than for chickens without additives. As in our experiment, there were trends that dietary additives led to a lower (p=0.07) number of lymphocytes [35].

Joint feeding with BC+CO increased the values for granulocytes, Hb, and Ht, which is probably associated with the manifestation of properties, primarily probiotic ones. The mechanism of its action may be explained by the fact that strong resistance to the concentrations studied was discovered previously in a study on the inhibitory action of umbelliprenin (CO) on mononuclear peripheral blood cells of both human and mouse origin (IC_50_ in the range from 713 to 6651 μg/mL) [16].

The addition of CO to the diet reduced the content of GLU in the serum of broilers, as well as that of cholesterol, and increased TG (similar to BC), CREAT, UA, and iron.

The reduction in GLU when CO was added to the diet of broilers was probably because CO is currently considered as a potential antidiabetic drug [36]. Among the derivatives of CO, new double inhibitors of α-glucosidase and α-amylase have been identified, and some compounds show high activity that stimulates GLU consumption [37]. Moreover, COs have been discovered that reduce the content of lipids in the body [38] and inhibit absorption of urate (osthol) [39] that likely contributes to the reduction of lipids and the increase of UA and CREAT in the CO group. The role of COs in the mobilization of iron by its recovery and chelation in plants has recently been confirmed [40]: An analogous mechanism could occur in the body of broilers in our study.

The BC+CO group was characterized by a decrease in BIL with an increase of UA and minerals (Fe, P). The mechanism of reducing BIL could be associated with the detection of BIL oxidase activity in *Bacillus* strains [41], which may have affected its content in the serum of broilers. As for the possible mechanisms of increasing UA and minerals, they could be analogous to that described above (for the CO group). Our findings are consistent with those of Gong *et al*. [28] who introduced the probiotic strain BC to broiler feed and recorded significantly reduced levels of TC and TG.

The activity of the endogenous enzyme ALT in the CO group was higher than for other groups. This reaction can be explained by the fact that CO is a toxic substance while the safety of its use with food has been confirmed by several scientists [12]. CO is not DNA reactive and it has been shown that species-specific toxicity to the liver is associated with the pharmacokinetics of CO metabolism [42].

The activity of AST in all experimental groups decreased significantly, as did that of γ-glutamyltransferase in groups with CO. This is probably due to the hepatoprotective properties which COs and *Bacillus* probiotics possess, as confirmed by studies by Tian *et al*. [43], Adorian *et al*. [33], and Abudabos *et al*. [44].

The inclusion of BC and BC+CO in the poultry diets contributed to an increase in the number of chemical elements in hepatic tissue (Ca, K, Mg, Mn, Si, and Zn for BC+CO; Ca, Fe, Ni, As, B, Co, Cr, and I for BC; As, B, Co, Cr, and I for CO). There is limited information in the literature about the effects of BC or CO on the mineral composition of the liver or muscle tissue of broilers. Therefore, our study was conducted to obtain additional information on this topic. However, the literature about micro- and macronutrients in the liver of farm animals [45] shows the dependence of substance concentrations on the environment and feed factors.

In the pectoral muscles, the addition of BC+CO to the diet contributed to an increase of more chemical elements (Ca, Na, Co, Cu, Fe, Mn, Ni, and Zn) than the addition of BC (Co, Ni, and Zn) and CO alone (Ca, Na, Co, and Ni). In addition, most chemical elements in the thigh muscles decreased, except for K, Mg, P, and B, the levels of which hardly changed.

The previous studies reported the effect of a diet supplemented with fatty seeds on the mineral composition of the pectoral muscles of broilers. For example, high concentrations of Fe found in the muscles could be associated with high levels of Fe in the blood [46]. The findings of our study are consistent with those of the previous studies, as the Fe content in the blood of the BC+CO group was high, as was the content in the muscles.

Other authors have also noted that probiotic substances increase the concentration of all minerals in the serum [47].

Given that COs are contained in extracts from many plants, we assume that similar studies related to the inclusion of plants in rations for broilers may explain our findings to some extent. For example, in a study assessing different levels of *Boswellia serrata* ethereal extract supplementation in the diet of broiler chickens, increased Ca levels were found in chest and thigh muscles and the liver, as well as Mg in thigh muscles and the liver [48]. The addition of *B. serrata* reduced the Cu content in the chest and thigh muscles and the liver and the retention of Zn in the thigh muscles. In our case, an analogous effect was observed for Ca, Mg, and Zn in the liver.

Feeding with coriander and rosemary supplements showed no significant differences in K and Fe in broiler meat, while feeding with rosemary was characterized by the highest percentages for Na, Mg, and Ca [49], which is also consistent with our findings. It was previously noted that Fe is the dominant metal in the liver, and Zn is the dominant metal in the thigh and pectoral muscles of broilers [50], which is also consistent with our results.

In our opinion, the possible mechanisms of BC action in the body that provides the change in the chemical composition of hepatic and muscular tissue may be its production of bacteriocins, suppression of virulence gene expression, competition for the adhesion of substances, production of lytic enzymes and antibiotics, immunostimulation, competition for nutrients and energy, and organic acid production [51-55]. Possible mechanisms of CO action in the bird may be the manifestation of antibacterial and antioxidant properties [56,57], modulation of the intestinal microbiome and lipid metabolism [58], and hepatoprotective and anti-aggregative properties [59,60].

## Conclusion

The study showed that the inclusion of CO and CO+BC in the diet of broilers improves their growth rates and reduces FC in broilers with the absence of mortality during the experiment.Using the compounds cause adverse reactions in the body: Decrease in leukocytes (BC), increased metabolism of proteins in the muscular tissue, TG (CO), and uric acid in the blood (CO and BC+CO). The inclusion of BC+CO in the diet contributes to increases of a greater number of chemical elements in the liver (Ca, K, Mg, Mn, Si, and Zn) and the pectoral muscles (Ca, Na, Co, Cu, Fe, Mn, Ni, and Zn) of broilers with decreases in thigh muscles.

## Authors’ Contributions

GD was responsible for conception and design of the study. SR coordinated the analysis. OK was responsible for the conclusive and final remarks. GD did the final editing and approval along with researchers. All authors read and approved the final manuscript.
